# Target-Specific Electrochemical Sensing of Pipecolic Acid via Molecular Imprinting

**DOI:** 10.3390/polym18091066

**Published:** 2026-04-28

**Authors:** Nihal Ermiş

**Affiliations:** 1 Department of Fundamental Sciences, Faculty of Engineering and Natural Sciences, Samsun University, 55420 Samsun, Türkiye; nihal.ermis@samsun.edu.tr; 2 Department of Biomedical Engineering, Faculty of Engineering and Natural Sciences, Samsun University, 55420 Samsun, Türkiye

**Keywords:** pipecolic acid, molecularly imprinted polymer, electrochemical sensor, polypyrrole, differential pulse voltammetry

## Abstract

Pipecolic acid (PA) is an important biomarker associated with peroxisomal and neurological disorders, necessitating the development of rapid, selective, and cost-effective detection methods beyond conventional chromatographic techniques. In this study, a molecularly imprinted electrochemical sensor (PA-MIP/Au) was developed for the selective determination of PA. The sensor was fabricated by electropolymerizing pyrrole on a gold electrode in the presence of PA as a template, followed by template removal to create specific recognition cavities. The electrochemical behavior and analytical performance were evaluated using cyclic voltammetry (CV), electrochemical impedance spectroscopy (EIS), and differential pulse voltammetry (DPV) in a ferri/ferrocyanide redox system. The sensor exhibited a linear response over 5–100 µM, with a detection limit of 1.05 µM. This range covers the reported physiological plasma concentrations of pipecolic acid (0.7–2.6 µM) and extends to elevated levels observed in pathological conditions, thereby demonstrating its suitability for clinical and biochemical monitoring applications. The sensor also demonstrated high selectivity against structurally similar amino acids, good repeatability, reproducibility, and stability, retaining over 87% of its initial response after 28 days. Recovery studies in spiked artificial plasma samples yielded values between 97.2% and 98.4%, confirming its applicability in complex matrices. Overall, the proposed sensor offers a simple, rapid, and cost-effective alternative for PA determination with potential for clinical and point-of-care applications.

## 1. Introduction

Pipecolic acid (PA) is a six-membered heterocyclic imino acid derived from lysine metabolism and exists in two enantiomeric forms: D-pipecolic acid and L-pipecolic acid. In humans, the L-isomer is the physiologically relevant form. It plays an integral role in the L-pipecolate pathway, a minor but significant route for lysine catabolism, particularly in the brain and liver. In this pathway, L-PA is oxidized by L-pipecolate oxidase, a peroxisomal enzyme, to α-aminoadipate semialdehyde (AASA), linking PA metabolism to peroxisomal function and redox homeostasis [1,2].

The clinical relevance of pipecolic acid lies primarily in its diagnostic role as a biomarker of peroxisomal dysfunction. Disruptions in the pathway are closely tied to peroxisomal biogenesis disorders (PBDs), such as Zellweger syndrome, in which peroxisomal enzyme deficiency leads to L-PA accumulation in plasma and cerebrospinal fluid [3]. Elevated plasma or cerebrospinal fluid levels of L-PA are characteristic of Zellweger spectrum disorders, neonatal adrenoleukodystrophy, and other PBDs [4]. In addition, elevated levels have been consistently associated with pyridoxine-dependent epilepsy (PDE), where impaired lysine catabolism due to *ALDH7A1* deficiency leads to PA accumulation in plasma and cerebrospinal fluid; thus, PA has been identified as a secondary biochemical marker supporting the diagnosis of PDE and related epileptic encephalopathies [5,6]. Moreover, elevated PA levels have been linked to hepatic encephalopathy and chronic liver diseases, suggesting that it may reflect broader hepatic and peroxisomal metabolic impairment [7]. Thus, PA serves as a metabolic bridge between lysine degradation, peroxisomal function, and neurological health, making it a target of growing biomedical interest.

Accurate detection of pipecolic acid (PA) requires sensitive, selective, and reliable analytical methods due to its low endogenous concentrations and structural similarity to other amino acids. Various chromatographic and spectrometric techniques have been developed for biological and environmental matrices. Early analytical efforts quantified PA using cation-exchange chromatography with post-column ninhydrin or o-phthalaldehyde (OPA) derivatization. While these systems provided foundational data for clinical biochemistry, they suffer from long runtimes and poor selectivity for cyclic secondary amines [2]. HPLC remains widely used for PA detection, often after pre-column derivatization to enhance UV or fluorescence response. Derivatizing agents such as ninhydrin, dansyl chloride, or phenylisothiocyanate enable detection at low micromolar levels. Moulin et al. (2002) demonstrated a microwave-assisted ninhydrin derivatization method to shorten reaction times and improve sensitivity [8]. LC–MS/MS has become the gold standard for PA quantification in clinical laboratories due to its superior selectivity and throughput. It enables stereospecific quantification of L- and D-PA using chiral stationary phases [9]. The method by Semeraro et al. (2015) achieved simultaneous analysis of PA and related metabolites in plasma without derivatization, achieving limits of detection (LOD) below 0.01 µM [10]. GC–MS after propyl-chloroformate derivatization offers rapid quantitation for plant and biological samples. Yu et al. (2020) developed a one-step protocol with minimal sample preparation, providing high reproducibility and sensitivity (LOD ~0.05 µM) [11] (Table 1).

Compared to these conventional chromatographic and spectrometric approaches, a molecularly imprinted electrochemical sensor (MIP-ECS) offers significant advantages for pipecolic acid determination. MIP-ECS platforms combine molecular selectivity with rapid, low-cost, and label-free detection, eliminating the need for derivatization, complex sample preparation, or expensive instrumentation. Once optimized, the sensor can achieve micromolar sensitivity, operate in complex matrices, and enable portable, real-time monitoring, making it a promising alternative to LC–MS/MS or HPLC for clinical and point-of-care applications.

In this study, a molecularly imprinted electrochemical sensor (MIP-ECS) was developed for the selective and sensitive detection of pipecolic acid. The sensor was fabricated by electropolymerizing pyrrole in the presence of pipecolic acid as a template molecule on a gold electrode. Polypyrrole was selected as the functional monomer due to its excellent electrochemical properties, ease of electropolymerization, and ability to form stable thin films on electrode surfaces, as widely reported in molecularly imprinted electrochemical sensor studies. The template molecule was subsequently removed by treatment with an acidic solution, thereby forming specific recognition cavities within the polymer matrix. The rebinding of the target molecule was monitored through differential pulse voltammetry (DPV), electrochemical impedance spectroscopy (EIS) and cyclic voltammetry (CV) using a ferri/ferrocyanide redox probe. The sensor exhibited high sensitivity within the micromolar concentration range relevant to physiological and pathological levels, demonstrating the potential of MIP-based electrochemical platforms as a rapid, low-cost, and portable alternative to chromatographic and mass spectrometric methods for pipecolic acid determination.

## 2. Materials and Methods

### 2.1. Reagents

L-pipecolic acid (PA), pyrrole (Py, ≥99%), potassium chloride (KCl), potassium ferricyanide [K_3_Fe(CN)_6_], potassium ferrocyanide [K_4_Fe(CN)_6_], sodium chloride (NaCl), hydrochloric acid (HCl) were purchased from Sigma-Aldrich (St. Louis, MO, USA) and used without further purification. Artificial plasma fluid was purchased from Biochemazone (Leduc, AB, Canada). Deionized (DI) water (18.2 MΩ·cm) was obtained from a Merck Millipore (Darmstadt, Germany) system. All solutions were prepared freshly before use.

### 2.2. Apparatus

Electrochemical experiments were carried out using a CH Instruments CHI660E electrochemical workstation (Austin, TX, USA) equipped with a conventional three-electrode cell consisting of a gold working electrode (Au, 3 mm diameter), a platinum wire counter electrode, and an Ag/AgCl (3 M KCl) reference electrode. Cyclic voltammetry (CV), differential pulse voltammetry (DPV), and electrochemical impedance spectroscopy (EIS) were performed using 5 mM [Fe(CN)_6_]^3−^/[Fe(CN)_6_]^4−^ in 0.1 M KCl as the redox probe. All potentials were reported versus Ag/AgCl at room temperature. SEM images were captured using a JEOL JSM-7001F (JEOL USA, Inc., Peabody, MA, USA) device, operating at an accelerating voltage of 15 kV, allowing for high-resolution visualization of the polymer surface structure.

### 2.3. Fabrication of the Imprinted and Non-Imprinted Electrodes

**Figure 1:**
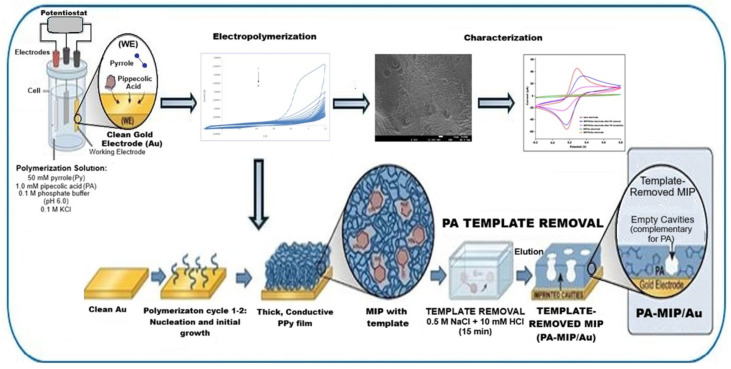
Schematic illustration of the fabrication procedure of the PA-MIP/Au electrode.

The process of fabricating the molecularly imprinted polymer (MIP) sensor based on electropolymerized polypyrrole is schematically illustrated in Figure 1. Before MIP fabrication, the Au electrode was polished with 0.05 µm alumina slurry, rinsed with DI water, and ultrasonicated in ethanol and water for 2 min each. Electrochemical cleaning was then performed in 0.5 M H_2_SO_4_ by cycling the potential between −0.2 V and +1.5 V (50 mV s^−1^, 20 cycles). The polymerization solution consisted of 50 mM pyrrole and 1.0 mM pipecolic acid prepared in 0.1 M phosphate buffer (pH 6.0) containing 0.1 M KCl as supporting electrolyte. The molecularly imprinted polymer (MIP) film was prepared by electropolymerizing pyrrole on the cleaned gold electrode in the presence of L-pipecolic acid as the template in 0.1 M KCl supporting electrolyte (pH 6.0, 0.1 M phosphate buffer). The electropolymerization was conducted between −0.2 V and +1.2 V at a scan rate of 100 mV s^−1^ for 20 cycles at room temperature (25 ± 2 °C).

After electropolymerization, the electrode was rinsed thoroughly with deionized water and gently air-dried at room temperature (25 ± 2 °C). Template removal was performed in 0.5 M NaCl + 10 mM HCl solution under static conditions at room temperature for 15 min. After template removal, the electrode was rinsed with deionized water and equilibrated in phosphate buffer (pH 7.0) for 10 min before measurements. The elution of PA was monitored by EIS and CV measurements on the redox probe, where a decrease in charge-transfer resistance (Rct) and an increase in current associated with the PA-eluted state indicated successful opening of the imprinted pores [12,13]. The resulting electrode was denoted as PA-MIP/Au. A non-imprinted polymer (NIP) electrode was fabricated under the same conditions without adding PA to the polymerization solution, serving as a control electrode and denoted as NIP/Au. All electrochemical measurements were conducted at room temperature using freshly prepared redox probe solutions.

For quantitative analysis, the rebinding of PA (5–100 µM) was monitored by DPV (0.6–0.95 V, pulse amplitude = 50 mV, step = 5 mV) and EIS (10^5^–0.1 Hz, AC amplitude = 10 mV). The change in peak current (ΔI_p_) or charge-transfer resistance (ΔR_ct_) was correlated with PA presence to construct curves for calibration and characterization. The sensor’s selectivity was evaluated against structurally similar amino acids (proline, lysine, glycine) and ascorbic acid and the practical applicability of the sensor was tested through recovery studies in spiked artificial plasma samples.

## 3. Results

### 3.1. Electropolymerization of Pyrrole

**Figure 2:**
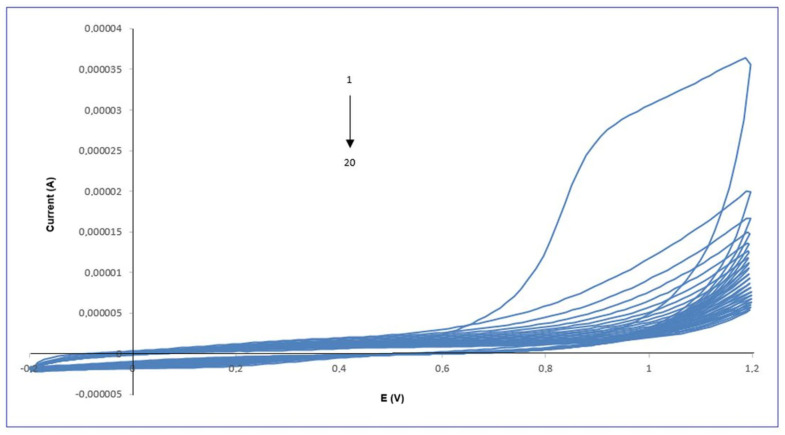
Cyclic voltammograms recorded during the electropolymerization of pyrrole on the Au electrode (scan rate: 100 mV s^−1^, potential range: −0.2 to +1.2 V, 20 cycles).

The electropolymerization of pyrrole (Py) on the gold electrode surface was monitored by cyclic voltammetry (CV), and the resulting voltammograms are shown in Figure 2. The polypyrrole (PPy) film was electropolymerized onto the cleaned gold electrode surface by cyclic voltammetry in a mildly acidic buffered medium. The polymerization solution contained 50 mM pyrrole and 1.0 mM pipecolic acid, prepared in 0.1 M phosphate buffer (pH 6.0) supplemented with 0.1 M KCl as the supporting electrolyte. The potential was cycled between −0.2 V and +1.2 V at a scan rate of 100 mV s^−1^.

The potential was cycled between −0.2 V and +1.2 V to induce oxidative polymerization, resulting in the progressive growth of a conductive polypyrrole (PPy) film. As shown in Figure 2, the anodic current increased gradually with the first cycle, particularly in the region of +0.9 V, indicating oxidation of pyrrole monomers and the formation of a thicker PPy layer on the electrode surface. This characteristic increase in oxidation current is consistent with the well-documented electropolymerization behavior of pyrrole, as reported in previous studies [14,15].

With progressive cycling, a systematic suppression of the anodic oxidation peak became evident, indicative of surface passivation arising from the nucleation and growth of an electropolymerized film. After the electropolymerization step, the voltammetric current decreased sharply, which is attributed to the impaired charge-transfer kinetics and restricted diffusion of redox probes toward the electrode interface due to the increasing thickness and insulating nature of the deposited polymer layer [12,16].

### 3.2. Characterization of the Electrode Surfaces

**Figure 3:**
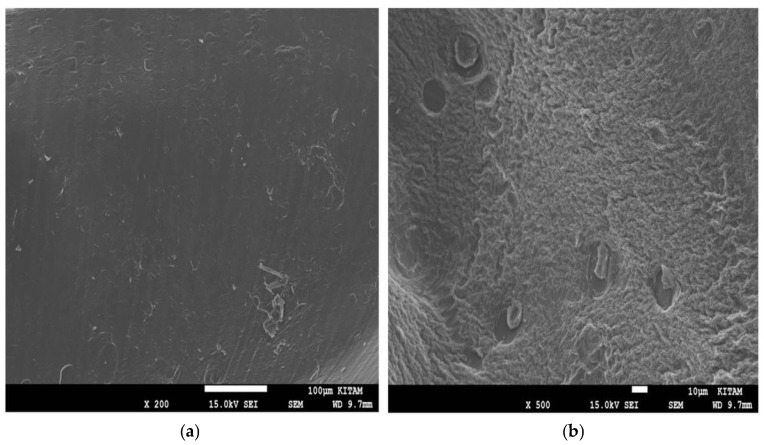
SEM images of the polymer-modified electrodes: (**a**) non-imprinted polymer on the gold electrode (NIP/Au) (**b**) pipecolic acid-imprinted polymer electrode (PA-MIP/Au) after template removal.

Scanning electron microscopy (SEM) was employed to investigate the surface morphology of the modified electrodes and to evaluate the structural differences between the non-imprinted polymer (NIP) and molecularly imprinted polymer (MIP) layers formed on the gold electrode surface (Figure 3). The SEM image of the NIP/Au electrode shows a relatively smooth and compact polymer film without noticeable cavities, indicating homogeneous polymer growth in the absence of the template molecule (Figure 3a). In contrast, the PA-MIP/Au electrode exhibits a rough, porous morphology with several microcavities distributed across its surface (Figure 3b). These cavities are attributed to the removal of pipecolic acid from the polypyrrole matrix, indicating morphological features consistent with imprinted cavities.

### 3.3. Electrochemical Behavior of the PA-MIP/Au Electrode

The electrochemical behavior of the PA-MIP/Au and NIP/Au electrode was systematically investigated using CV, EIS, and scan rate studies to elucidate the charge transfer characteristics and binding-induced changes at the electrode interface.

**Figure 4:**
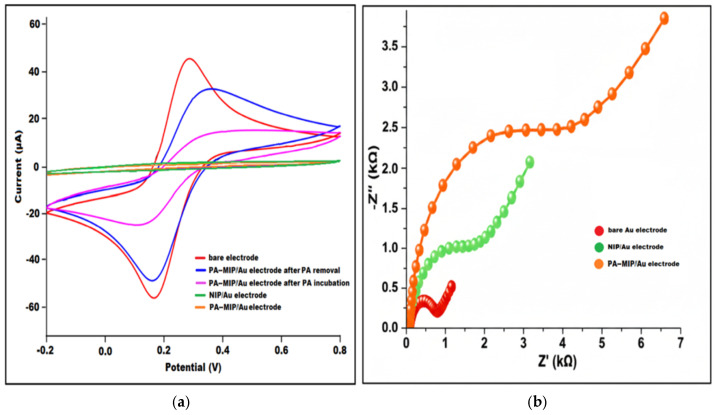
(**a**) Cyclic voltammograms and (**b**) EIS spectra of different modified electrodes in the solution of 0.1 M KCl containing 5 mM [Fe(CN)_6_]^3−/4−^.

The cyclic voltammograms illustrate the electrochemical behavior of bare Au electrode, MIP- and NIP-modified Au electrodes in 5 mM [Fe(CN)_6_]^3−^/[Fe(CN)_6_]^4−^ in 0.1 mol/L KCl solution as redox probe (Figure 4a). The bare Au electrode (red) exhibits the highest anodic and cathodic peak currents, consistent with its unobstructed electron transfer surface. However, after polymerization, the peak current decreased significantly because of constraint of electron transfer caused by polymer formation (orange). PA-imprinted polymeric film hindered the occurrence of redox reaction, which caused current reduction. Following PA removal (blue), the specific cavities facilitate the redox reaction, resulting in a current increase. After incubation with PA, the PA-MIP/Au electrode (magenta) displays a substantial reduction in peak currents, confirming successful rebinding of PA molecules into the imprinted sites and the associated hindered diffusion of the redox species—a characteristic response reported for MIP-based sensing layers.

In contrast, the NIP/Au (green) and PA-MIP/Au (orange) electrodes present only minimal current variations, reflecting the absence of specific recognition cavities and demonstrating the nonspecific, diffusion-limited behavior of the polymers. Overall, the significant current suppression observed after PA rebinding on the MIP electrode highlights the selective binding capability of the imprinted polymer structure, in agreement with previous MIP-based electrochemical sensor studies [17,18].

The Nyquist plots of the bare Au, NIP/Au, and PA-MIP/Au electrodes are presented in Figure 4b. In all spectra, the semicircle observed in the high-frequency region corresponds to the charge transfer resistance (R_ct_), which reflects the interfacial electron transfer kinetics of the redox probe. The bare Au electrode exhibited the lowest R_ct_ value (0.92 kΩ), indicating rapid electron transfer at the clean gold surface. After modification with the non-imprinted polymer (NIP), R_ct_ increased markedly to 2.63 kΩ, confirming the formation of a partially insulating polymer film that hinders charge transfer. A further significant increase in R_ct_ was observed for the MIP/PA/Au electrode (≈4.8–5.0 kΩ). The nearly fivefold increase compared to bare Au demonstrates the successful formation of a dense imprinted polymer layer, which restricts diffusion of the redox species and increases interfacial resistance. This drastic rise in impedance supports the formation of the grafting of the molecularly imprinted polymer matrix, effectively hindering the diffusion of redox probe ions to the electrode surface [19,20].

**Figure 5:**
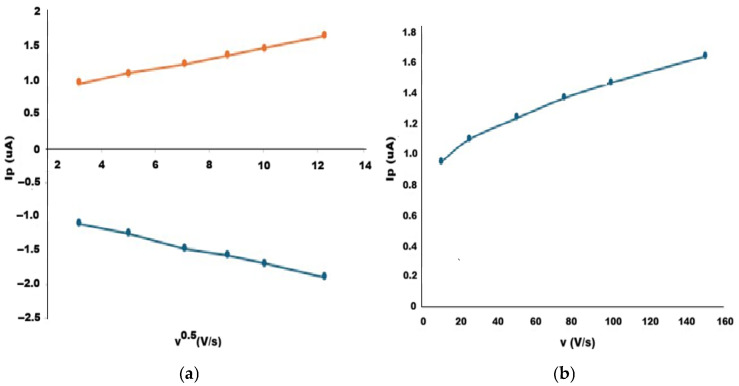
Scan rate study of (**a**) the bare electrode and (**b**) the MIP-modified electrode in 5 mM K_3_Fe(CN)_6_/0.1 M KCl solution.

The electrochemical behavior of the bare and the modified electrodes were also investigated by cyclic voltammetry using 5 mM K_3_Fe(CN)_6_ in 0.1 M KCl as a redox probe. The potential was scanned between 0 V and +0.6 V at different scan rates ranging from 10 to 150 mV s^−1^. The relationship between the current values (Ip) and scan rates were investigated (Figure 5). The electrochemical behavior and the electroactive surface area of the bare and MIP electrodes have been explained by the Randles–Sevcik equation for a reversible system at 25 °C:

Ip = (2.69 × 10^5^) n^3/2^ A D^1/2^ c ν^1/2^

where Ip is the peak current (A), n is the number of electrons transferred, D is the diffusion coefficient of the redox probe (cm^2^ s^−1^), c is the concentration (mol cm^−3^), ν is the scan rate (V s^−1^), and A is the electroactive surface area (cm^2^).

To analyze the charge transfer mechanism of the bare electrode, the peak current values (Ip) were plotted against the square root of the scan rate (ν^1/2^) (Figure 5a). A linear relationship was obtained over the studied scan rate range, indicating that the electrochemical process is diffusion-controlled. This behavior suggests that the electrode response is governed by the diffusion of electroactive species from the bulk solution to the electrode surface, consistent with the Randles–Ševčík equation. The modified electrode exhibited a direct linear relationship between the peak current (Ip) and the scan rate (ν) (Figure 5b). This type of dependence is more related to the characteristic of a surface-controlled (adsorption-controlled) process. This behavior is attributed to the presence of the polymeric film, which restricts diffusion of the redox probe and confines the electron transfer process to the electrode interface. The analyte itself modulates the electrochemical response by occupying the imprinted cavities and hindering access of the redox probe [21].

In addition, the electroactive surface areas of the bare and MIP-modified electrodes were also calculated using the Randles–Ševčík equation. The effective surface areas were estimated as 0.031 cm^2^ for the bare electrode and 0.022 cm^2^ for the MIP electrode. The calculated electroactive surface area for the MIP electrode represents an apparent value due to the deviation from purely diffusion-controlled behavior. The decrease in electroactive surface area after MIP modification confirms the successful formation of the polymeric film on the electrode surface. The imprinted layer partially hinders electron transfer of the redox probe, resulting in a lower apparent active area. The observed shift from diffusion-controlled behavior at the bare electrode to a surface-controlled process at the modified electrode provides strong evidence for the successful formation of the molecularly imprinted film. This change indicates that the electrochemical response is predominantly governed by binding events occurring within the imprinted cavities of the polymer layer.

### 3.4. Optimization of the Molecular Imprinting Procedure

#### 3.4.1. Monomer/Template Ratio

**Figure 6:**
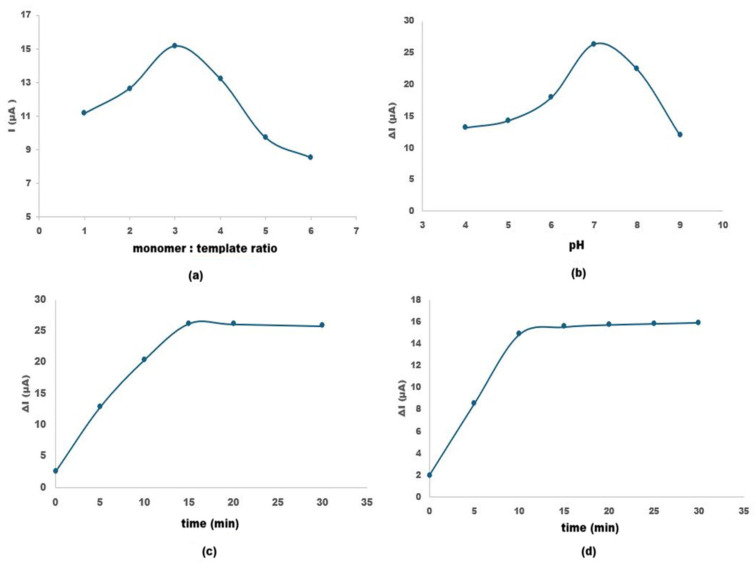
Optimization of experimental parameters for the PA-MIP sensor: (**a**) effect of monomer-to-template ratio on peak current response; (**b**) influence of solution pH on ∆I; (**c**) effect of elution time on ∆I; (**d**) effect of incubation time on ∆I (in 5 mM [Fe(CN)_6_]^3−^/^4−^ containing 0.1 M KCl).

Polymeric film formation is closely governed by the interactions between the monomer and the template molecule; therefore, optimizing the monomer-to-template ratio is essential. This ratio is one of the key parameters determining the stability and efficiency of the resulting polymeric film. Insufficient monomer-to-template ratios limit the effective participation of functional groups in the polymer matrix, leading to poorly defined recognition sites, whereas excessively high ratios result in disordered monomer packing and a consequent loss of selectivity [22]. Therefore, different monomer-to-template ratios (1:1, 2:1, 3:1, 4:1, 5:1, and 6:1) were systematically investigated (Figure 6a). After polymerization, the prepared MIP sensors were eluted to remove the template molecules, and their electrochemical responses were recorded by cyclic voltammetry (CV). The peak current values obtained after template removal were compared to evaluate the effect of monomer-to-template ratio on the imprinting efficiency. As the pyrrole-to-pipecolic acid ratio increased from 1:1 to higher values, the current response initially increased, reflecting the formation of a greater number of imprinting sites and enhanced accessibility of the redox probe after template removal. However, at excessively high monomer contents, the current value started to decrease, which can be attributed to disruption of the polymeric network and the generation of irregular, poorly defined cavities rather than well-shaped recognition sites. Thus, the 3:1 pyrrole-to-pipecolic acid ratio, at which current value reached its maximum, was selected as the optimal composition for subsequent sensor fabrication, providing a suitable balance between efficient imprinting and structural integrity of the film.

#### 3.4.2. The pH of the Medium

The electrolyte pH plays a crucial role in governing both the electrochemical response and the structural stability of the molecularly imprinted membrane on the electrode surface. Thus, careful optimization of the pH is required to prevent degradation of the imprinted polymer layer and to maintain a consistent and reproducible current signal during analysis.

The effect of the solution pH on the current response of the pipecolic acid-imprinted polypyrrole sensor was evaluated between pH 4.0 and 9.0 (Figure 6b). The current response increased gradually from pH 4.0 to reach a maximum at pH 7.0 (≈26 µA), followed by a noticeable decrease at higher pH values. The enhanced signal at neutral pH can be attributed to the favorable ionization state of pipecolic acid and optimal protonation level of the polypyrrole matrix, which promotes stronger template–polymer interactions and more efficient charge transfer. At lower pH values, excessive protonation of the PPy backbone may reduce conductivity and alter binding interactions, while at alkaline pH, deprotonation of both the analyte and polymer matrix weakens electrostatic interactions and decreases recognition efficiency. Similar pH-dependent behavior has been reported for polypyrrole-based molecularly imprinted electrochemical sensors, where maximum responses are typically observed near physiological pH due to balanced protonation and optimal binding affinity [23]. Therefore, pH 7.0 was selected as the optimal working condition for subsequent analytical measurements.

#### 3.4.3. Optimization of Elution Time

Optimizing elution time is a crucial parameter to ensure complete removal of pipecolic acid from the polypyrrole matrix, thereby vacating the imprinted cavities for subsequent rebinding. The effect of elution time was investigated by monitoring the recovery of the electrochemical signal at different intervals ranging from 0 to 30 min, indicating progressive removal of the embedded template molecules and restoration of accessible binding cavities (Figure 6c). Following each elution step, the electrode was thoroughly rinsed with deionized water and placed into a 5.0 mM [Fe(CN)_6_]^3−/4−^ redox probe solution containing 0.1 M KCl. The recovery of the electrochemical signal was monitored using CV within a potential range of −0.2 V to +0.6 V. The change in the peak current was calculated by subtracting the initial current (before elution) from the current obtained after each specific elution period. The time at which the ΔI reached a maximum and stabilized was identified as the optimum elution time, ensuring the effective removal of the template from the imprinted cavities.

As presented in Figure 6c, the current response significantly increased within the first 10 min, indicating a rapid release of the template molecules from the surface. The increase in ΔI reflects the progressive removal of template molecules and restoration of electron transfer pathways between the redox probe and electrode surface. The maximum change in peak current occurred at 15 min, after which the signal reached a steady state. This stabilization suggests that all accessible imprinted sites were effectively cleared. Prolonged elution beyond 15 min did not yield further improvement and could potentially lead to the degradation of the polymer’s structural integrity. Consequently, 15 min was established as the optimum elution time for achieving high sensitivity and reproducibility in the sensing process.

#### 3.4.4. Optimization of Incubation Time

The incubation time plays a critical role in determining the binding efficiency of analyte to the imprinted cavities of the MIP-modified electrode. An adequate incubation period ensures effective diffusion and rebinding of PA molecules into the recognition sites, whereas unnecessarily prolonged incubation does not significantly improve the analytical response and may reduce the practical applicability of the sensor. Therefore, incubation time was optimized under the previously established optimum conditions.

After the optimized elution step (15 min), the MIP electrode was incubated with 50 µM pipecolic acid solution for different time intervals (0–30 min) (Figure 6d). Following each incubation step, the electrode was rinsed with deionized water to remove loosely bound molecules and transferred into a 5.0 mM [Fe(CN)_6_]^3−^/^4−^ solution containing 0.1 M KCl. Cyclic voltammetry (CV) measurements were performed within the potential range of −0.2 V to +0.6 V. The change in peak current (ΔI) was calculated by subtracting the current obtained after incubation from the current measured immediately after elution.

As presented in Figure 6d, the increase in ΔI corresponds to the rebinding kinetics of pipecolic acid into the imprinted cavities, leading to gradual surface blocking and signal modulation. The results indicate that 10 min is sufficient for effective rebinding of 50 µM pipecolic acid into the specific cavities of the MIP layer. Therefore, 10 min was selected as the optimal incubation time for subsequent analytical studies, as it provides the highest and most stable signal while maintaining a short analysis time.

### 3.5. Electroanalytical Performance of the PA-MIP/Au Sensor

**Figure 7:**
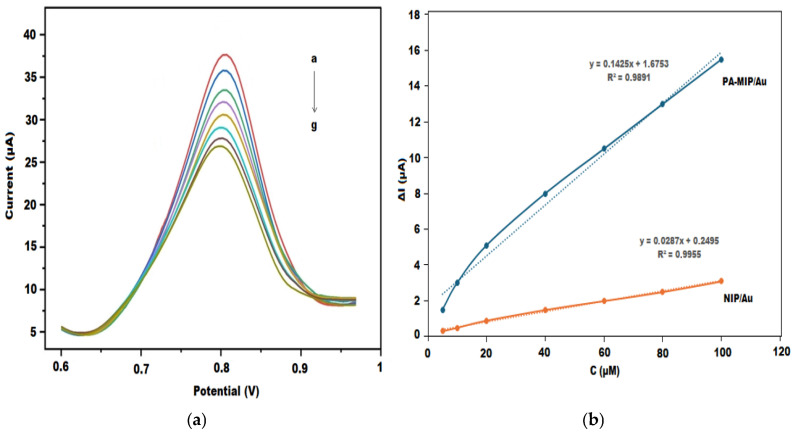
(**a**) DPV responses of the PA-MIP/Au electrode after incubation with increasing concentrations of pipecolic acid (a to g; 0, 5, 10, 20, 40, 60, 80, and 100 µM PA); (**b**) Calibration plots of PA-MIP/Au and NIP/Au electrodes.

The analytical performance of the PA-MIP/Au sensor was evaluated in a 5 mM [Fe(CN)_6_]^3−^/^4−^ redox probe solution using the DPV technique after incubation at different concentrations of pipecolic acid (PA) (Figure 7a). As shown in the voltammograms, the peak current gradually decreased with increasing PA concentration, indicating progressive blockage of the imprinted cavities and hindered electron transfer at the electrode interface.

The analytical signal was calculated as ΔI = I_0_ − I, where I_0_ represents the peak current of the blank solution and I corresponds to the peak current measured after PA incubation. A linear relationship between ΔI and PA concentration was obtained in the range of 5–100 µM, with the regression equation: ΔI(μA) = 0.1425C(μM) + 1.6753 and a correlation coefficient of R^2^ = 0.9891 (Figure 7b).

The limit of detection (LOD) and limit of quantification (LOQ) were calculated according to LOD = 3σ/S and LOQ = 10σ/S, where σ is the standard deviation of the blank signal and S is the slope of the calibration curve. The LOD and LOQ values of the PA-MIP/Au sensor were determined to be 1.05 µM and 3.51 µM, respectively.

For comparison, a non-imprinted electrode (NIP/Au) was fabricated under identical conditions. Although a linear response was also observed for NIP/Au (R^2^ = 0.9955), its slope was significantly lower (0.0287 µA/µM) and the approximately fivefold higher sensitivity demonstrates the successful formation of selective recognition sites within the imprinted polymer matrix.

### 3.6. Selectivity Study

**Figure 8:**
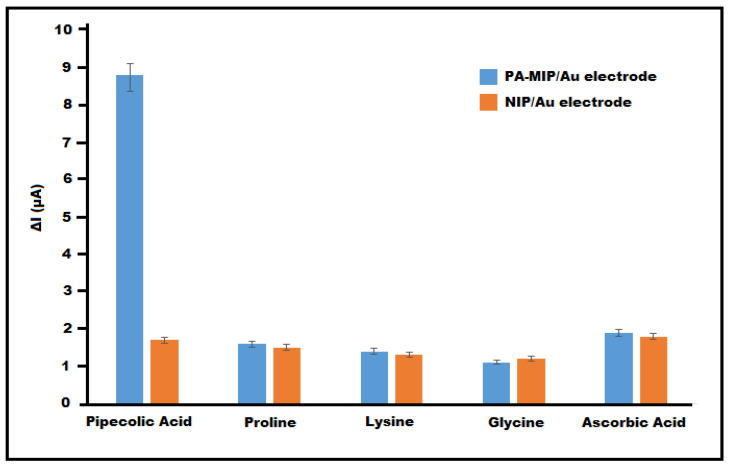
Selectivity comparison of PA-MIP/Au and NIP/Au electrodes toward pipecolic acid and interfering compounds (n = 3).

The selectivity of the PA-MIP/Au sensor toward PA was evaluated by comparing its response to structurally related compounds, including proline, lysine, and glycine, as well as a common electroactive interferent, ascorbic acid (Figure 8). While the amino acids were selected due to their structural similarity and potential competitive binding, ascorbic acid was included to assess possible interference arising from its redox activity.

The measurements were performed under identical experimental conditions in a 5 mM [Fe(CN)_6_]^3−^/^4−^ solution using DPV. The analytical signal was calculated as ΔI = I_0_ − I after incubation in 50 µM solutions of each analyte.

The PA-MIP/Au electrode exhibited a ΔI value of 8.8 µA for 50 µM PA. In contrast, significantly lower responses were obtained for the interfering species: proline (1.6 µA), lysine (1.4 µA), glycine (1.1 µA), and ascorbic acid (1.9 µA). The marked difference in signal confirms the preferential rebinding of PA within the imprinted cavities.

The selectivity coefficient (k), defined as:

$$k=\frac{\Delta I_{PA}}{\Delta I_{interferent}}$$

was calculated to further quantify selectivity [24]. The k values were determined as 5.5 (proline), 6.28 (lysine), 8.0 (glycine), and 4.63 (ascorbic acid), demonstrating strong discrimination in favor of PA.

For comparison, the NIP/Au electrode produced relatively low and comparable ΔI responses for PA (1.7 µA) and the interfering compounds (1.2–1.8 µA), indicating predominantly non-specific adsorption.

Furthermore, the imprinting factor (IF), calculated from the ratio of calibration slopes:

$$IF=\frac{Slope_{MIP}}{Slope_{NIP}}=\frac{0.1425}{0.0287}\approx 4.97$$

confirms the successful formation of PA-specific recognition sites [25]. The nearly fivefold increase in sensitivity for PA-MIP/Au compared to NIP/Au demonstrates that the sensing mechanism is primarily governed by molecular imprinting rather than nonspecific interactions.

This result highlights that the selectivity of the imprinted sensor is primarily governed by molecular recognition rather than electrochemical activity. Overall, the results verify that the PA-MIP/Au sensor exhibits high selectivity, strong molecular recognition, and effective discrimination against structurally related compounds.

### 3.7. Repeatability, Reproducibility and Stability of the PA-MIP/Au Sensor

The repeatability of the PA-MIP/Au sensor was evaluated by performing five consecutive DPV measurements using the same electrode in 50 µM PA solution under identical experimental conditions. The obtained ΔI responses were highly consistent, yielding a relative standard deviation (%RSD) of 2.15%, indicating good operational repeatability and stable signal generation during successive measurements (Table S1).

The reproducibility of the fabrication procedure was assessed by preparing five independent PA-MIP/Au electrodes under the optimum synthesis conditions. The ΔI values recorded for 50 µM PA showed satisfactory agreement, with a %RSD of 2.86%, demonstrating that the electrode preparation protocol provides reliable and reproducible sensor performance (Table S1).

The storage stability of the PA-MIP/Au sensor was further investigated by storing the modified electrodes at 4 °C in a dry environment and measuring their response periodically over 28 days. The sensor retained 92.4% of its initial response after two weeks, with no significant deterioration in signal intensity. At the end of 28 days, the sensor retained 87.3% of its initial response. The slight decrease observed may be attributed to minor structural relaxation within the polymer matrix or partial loss of surface activity over time (Figure S1).

Overall, the PA-MIP/Au sensor demonstrates excellent repeatability, good fabrication reproducibility, and satisfactory storage stability, confirming its suitability for reliable electroanalytical applications.

### 3.8. Determination of Pipecolic Acid in Real Samples

To evaluate the practical applicability of the developed MIP-based electrochemical sensor, spiked artificial plasma samples were used to assess matrix tolerance and analytical reliability. The samples were diluted (1:5, *v*/*v*) with 0.1 M KCl prior to analysis to minimize matrix effects and provide a stable ionic environment for electrochemical measurements.

Known concentrations of pipecolic acid (10, 25, and 50 µM) were spiked into artificial plasma samples (Table 2). After incubation of the eluted MIP electrode in the spiked samples, DPV measurements were performed in 5.0 mM [Fe(CN)_6_]^3−^/^4−^ solution containing 0.1 M KCl and each measurement was performed in triplicate. The recovery values ranged between 97.2% and 98.4%, with relative standard deviation values below 2.5%, indicating acceptable accuracy and precision of the proposed MIP sensor in artificial plasma fluid. These results verify that the developed PA-MIP/Au sensor has potential for the determination of pipecolic acid in biological samples without requiring complicated pretreatment procedures.

## 4. Discussion

The results obtained in this study clearly demonstrate that the electropolymerized polypyrrole-based molecularly imprinted sensor provides a selective and sensitive platform for the determination of pipecolic acid (PA). Polypyrrole has been widely employed as a functional matrix in molecularly imprinted electrochemical sensors due to its ease of electropolymerization, good electrical conductivity, and ability to form stable thin films on electrode surfaces. In addition, the presence of nitrogen-containing functional groups in its structure enables non-covalent interactions with target molecules. In the present system, selective recognition of pipecolic acid is mainly attributed to hydrogen bonding and electrostatic interactions between the polymer matrix and the amine and carboxyl groups of the analyte, together with the shape complementarity of the imprinted cavities. These combined effects contribute to the formation of selective binding sites within the polymer layer [21,23]. The electrochemical characterization results obtained by CV and EIS confirm the successful formation of the imprinted polymer layer and the generation of specific recognition sites after template removal. The observed increase in charge transfer resistance (Rct) and decrease in peak current following polymerization, together with the recovery of the signal after template removal, are consistent with the typical behavior of MIP-based electrochemical systems. Importantly, the partial recovery of current after template removal and its subsequent decrease upon rebinding confirm that the sensing mechanism is governed by reversible binding within imprinted cavities rather than nonspecific adsorption. The sensing mechanism is predominantly governed by non-covalent interactions and steric blocking effects within the imprinted cavities. Upon rebinding of PA, the occupation of these cavities hinders the diffusion of the redox probe, resulting in a decrease in current response. These findings confirm the successful fabrication and effective operation of the MIP-based sensor and are in good agreement with the literature [20,23].

The scan rate studies further revealed a transition from diffusion-controlled behavior at the bare electrode to a surface-controlled process at the MIP-modified electrode. This finding indicates that the electrochemical response is governed by analyte binding within the imprinted cavities rather than bulk diffusion, supporting the successful imprinting of PA within the polymer matrix [13]. The decrease in electroactive surface area after modification further confirms the formation of a polymeric layer that partially blocks electron transfer pathways. Such behavior has been reported for MIP-based electrochemical sensors and also confirms the formation of a polymeric film that partially restricts electron transfer while enabling selective recognition [26].

Optimization studies showed that a monomer-to-template ratio of 3:1 provided the highest current response, indicating optimal cavity formation. At lower ratios, insufficient functional monomer limits binding site formation, whereas higher ratios lead to structural disorder and reduced selectivity. Similarly, the maximum current response was obtained at pH 7.0, which can be attributed to the optimal protonation state of both pipecolic acid and the polypyrrole matrix. This observation aligns with previous studies reporting that polypyrrole-based MIP sensors exhibit maximum performance near physiological pH due to balanced electrostatic interactions [23]. This pH-dependent behavior also provides indirect evidence for the nature of the interactions between pipecolic acid and the polypyrrole matrix. The variation in signal with pH suggests that protonation–deprotonation equilibria play a critical role, indicating that the recognition mechanism is mainly governed by hydrogen bonding and electrostatic interactions rather than covalent binding.

The developed PA-MIP/Au sensor exhibited a linear response over the concentration range of 5–100 µM, with a detection limit of 1.05 µM. Although chromatographic techniques provide significantly lower detection limits, they require complex instrumentation, sample derivatization, and extensive sample preparation [2,8,9,10,11]. In contrast, the proposed sensor offers a simpler, faster, and more cost-effective alternative while maintaining adequate sensitivity at the micromolar level, which is sufficient for many practical applications. Thus, the method represents a favorable balance between analytical performance and operational simplicity. In addition to its satisfactory sensitivity, the sensor demonstrated rapid kinetics, with an optimal elution time of 15 min and an incubation time of 10 min, indicating efficient template removal and fast analyte rebinding. Compared to chromatographic methods, which typically require longer analysis times, the proposed sensor enables significantly faster detection [2,8,11]. These features highlight its strong potential for rapid, practical and real-time analytical applications.

The comparable selectivity responses obtained at the MIP and NIP electrodes against structurally similar amino acids such as proline, lysine, and glycine indicate that this interaction is predominantly non-specific. The significantly higher response of MIP electrode toward PA compared to structurally similar compounds further supports the presence of specific recognition sites formed via non-covalent interactions, confirming that the binding mechanism is driven by molecular-level interactions rather than nonspecific adsorption. This observation highlights that the selectivity of the sensor is mainly governed by molecular recognition rather than electrochemical activity, further validating the imprinting strategy.

The practical applicability of the sensor was validated using artificial plasma samples, with recovery values ranging from 97.2% to 98.4% and RSDs below 2.5%. These results confirm that the sensor maintains high accuracy and precision even in complex matrices without requiring extensive sample pretreatment. Compared with LC–MS/MS and GC–MS methods, which often require derivatization and matrix cleanup steps, the proposed sensor offers a simpler analytical workflow.

Overall, the developed PA-MIP/Au sensor combines acceptable sensitivity, high selectivity, rapid response, and operational simplicity. Although its detection limit is higher than that of advanced spectrometric techniques, its advantages in terms of cost, portability, and ease of use make it a promising alternative for point-of-care and on-site monitoring applications. The results demonstrate that molecular imprinting remains a powerful strategy for designing selective electrochemical sensors for biologically relevant small molecules such as pipecolic acid.

## 5. Conclusions

In this study, a molecularly imprinted polypyrrole-based electrochemical sensor was successfully developed on a gold electrode surface for the selective determination of pipecolic acid. The MIP layer was synthesized by electropolymerizing pyrrole in the presence of pipecolic acid as the template molecule, yielding specific recognition cavities upon template removal. The fabricated sensor exhibited a sensitive and reproducible response to pipecolic acid using differential pulse voltammetry with a ferri/ferrocyanide redox probe. The sensor demonstrated satisfactory analytical performance, including sensitivity, selectivity, and stability, with a low detection limit and good repeatability. Furthermore, the applicability of the proposed sensor was confirmed through successful recovery studies in artificial plasma samples, indicating minimal matrix interference. These results suggest that the developed MIP-based electrochemical platform offers a simple, reliable, and cost-effective approach for determining pipecolic acid and may serve as a promising tool for future clinical or biochemical monitoring applications.

## Figures and Tables

**Table 1:** Comparison of reported analytical methods for the determination of pipecolic acid.

Method	Detection Principle	Matrix	Derivatizing	Limit of Detection (LOD)	Run Time	Ref.
Ion-exchange chromatography	Post-column ninhydrin colorimetry	Plasma	Required	1 μM	>40 min	[2]
HPLC-UV	Pre-column ninhydrin	Serum	Required	0.2 μM	25 min	[8]
Chiral LC-MS/MS	Chiral separations of enantiomers	Plasma	Not required	0.02 μM	12 min	[9]
LC-MS/MS	Electrospray ionization	Plasma, urine	Not required	0.01 μM	10–15 min	[10]
GC-MS	Propyl chloroformate derivatization	Plant, plasma	Required	0.05 μM	20 min	[11]

**Table 2:** Determination of pipecolic acid in spiked artificial plasma samples (n = 3).

Added (µM)	Found (µM) Mean ± SD	Recovery (%)	RSD (%)
10	9.72 ± 0.22	97.2	2.2
25	24.38 ± 0.44	97.5	1.8
50	49.21 ± 0.83	98.4	1.7

## Data Availability

The original contributions presented in this study are included in the article/Supplementary Materials. Further inquiries can be directed to the corresponding author.

## Supplementary Materials

The following supporting information can be downloaded at: https://www.mdpi.com/article/10.3390/polym18091066/s1, Table S1. Repeatability and reproducibility of the PA-MIP/Au sensor (n = 5); Figure S1. Stability of the PA-MIP/Au sensor over 28 days (n = 3).

## Abbreviations

The following abbreviations are used in this manuscript:

PA	Pipecolic Acid
MIP	Molecularly imprinted polymer
NIP	Non-imprinted polymer
DPV	Differential pulse voltammetry
