# Supplementary material for: Target-Specific Electrochemical Sensing of Pipecolic Acid via Molecular Imprinting
Source: Polymers (Basel). 2026 Apr 28;18(9):1066. doi: 10.3390/polym18091066 (PMC13165148; doi:10.3390/polym18091066)
Supplement: Supplementary file 1 [file polymers-18-01066-s001.zip › polymers-4269740-supplementary.pdf]

Target-Specific Electrochemical Sensing of Pipecolic Acid via Molecular Imprinting

Table S1. Repeatability and reproducibility of the PA-MIP/Au sensor (n=5).

Parameter	Measurement No	ΔI (μA)	Mean (μA)± SD (μA)	RSD (%)
Repeatability	1	8.53		
	2	8.70		
	3	8.83	8.80±0.19	2.15
	4	8.99		
	5	8.95		
**Parameter**	**Electrode No**	**ΔI (μA)**	**Mean (μA)± SD (μA)**	**RSD (%)**
Reproducibility	1	8.42		
	2	8.71		
	3	9.08	8.80±0.25	2.86
	4	8.95		
	5	8.84		

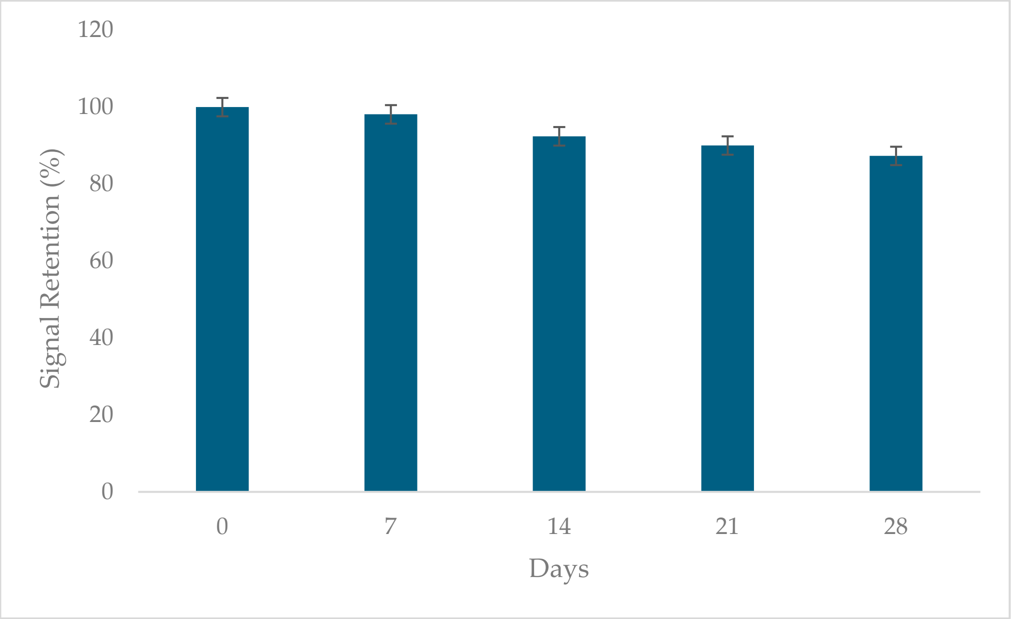

Figure S1. Stability of the PA-MIP/Au sensor over 28 days (n = 3).
